# Preliminary exploration of SKA1 expression in lung adenocarcinoma and its clinical significance: SKA1 expression in lung adenocarcinoma

**DOI:** 10.3724/abbs.2023243

**Published:** 2023-10-09

**Authors:** Wen Zeng, Yong Chen, Jun Liu, Zhen An, Hui Yan, Tao Sun

**Affiliations:** 1 Department of Scientific Research and Teaching the Central Hospital of Shaoyang City Shaoyang 422000 China; 2 Department of Oncology the Central Hospital of Shaoyang City Shaoyang 422000 China; 3 Department of Scientific Research the First Affiliated Hospital of Shaoyang University Shaoyang 422000 China; 4 Department of Hematology and Oncology Laboratory the Central Hospital of Shaoyang City Shaoyang 422000 China

The *SKA1* gene, a highly intricate and complex genetic locus of the human chromosome, is situated in the 17q21.31 region, contributing to its intricate and multifaceted nature [1]. Intriguingly, this gene encodes a variety of products that are capable of assembling into dynamic microtubule-associated complexes and play a pivotal role in ensuring accurate chromosomal segregation during mitosis [ 2– 4]. Beyond its well-established involvement in mitotic processes, the *SKA1* gene may also involve in the progression of various cancer types, adding an extra layer of complexity to its already intricately woven tapestry [2]. In fact, abnormal upregulation of SKA1 has been found in several tumor types, including stomach, lung, breast, ovarian, thyroid, and cervical cancers [2]. Despite these intriguing findings, there has been a notable dearth of research on the *SKA1* gene in lung cancer [5], with its expression level and biological function in this disease remaining enigmatic and shrouded in mystery. Therefore, the objective of our study is to elucidate the intricate and diverse involvement of SKA1 in lung cancer. This will be achieved through an extensive examination of its expression levels and clinical associations in lung adenocarcinoma tissue, as well as an exploration of its influence on patient outcomes, tumor progression, and metastasis using survival analysis and other methodologies. Additionally, we employed immunohistochemistry, a well-established and dependable technique for confirming disparities in SKA1 expression between lung adenocarcinoma and neighboring tissues. Ultimately, our study serves as a valuable reference for future investigations into the complex workings of the *SKA1* gene in lung cancer and potentially other forms of cancers.

**Figure 1:**
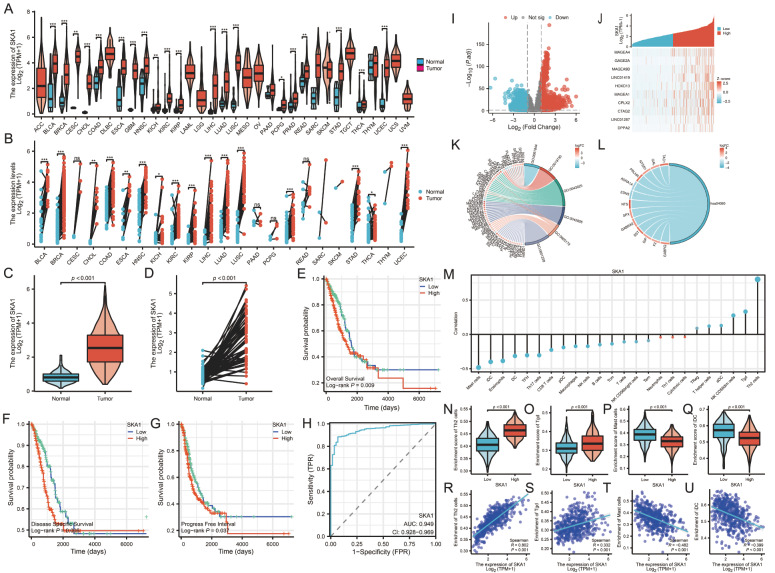
Figure 1 Correlation of *SKA1* expression with prognosis, diagnosis and immune infiltration of lung adenocarcinoma (A) SKA1 expression in different types of tumors compared with normal tissues in the TCGA database. (B) Comparison of SKA1 expression in different types of tumors with paired paracancerous tissues in the TCGA database. (C) Comparison of SKA1 levels in lung adenocarcinoma and normal tissues in the TCGA database. (D) Comparison of SKA1 levels in lung adenocarcinoma and paired paraneoplastic tissues in the TCGA database. (E) OS analysis of patients with lung adenocarcinoma. (F) DSS analysis of patients with lung adenocarcinoma. (G) Analysis of PFI in patients with lung adenocarcinoma. (H) ROC curves validate the role of SKA1 expression in the diagnosis of LUAD. (I) Volcano plot of DEGs. (J) Heatmap of the correlation between SKA1 expression and the top 10 DEGs. (K) GO analysis of DEGs. (L) KEGG analysis of DEGs. (M) Correlation between SKA1 expression and the relative abundance of 24 immune cell types. (N–Q) Comparison of immune infiltration levels of immune cells (including Th2 cells, Tgd cells, mast cells and iDCs) between the high and low SKA1 expression groups. (R–U) Correlation between the relative enrichment fraction of immune cells (including Th2 cells, Tgd cells, mast cells and iDCs) and the expression of SKA1.

This study screened the appropriate data from the publicly available lung adenocarcinoma sample dataset for analysis. We searched the dataset containing lung adenocarcinoma samples through TCGA databases and screened the data according to the following criteria: a) samples from tissue or blood samples of patients with lung adenocarcinoma; b) complete SKA1 expression data and clinical characteristic data; and c) more than 50 samples. We meticulously evaluated the expression levels of the *SKA1* gene in both lung adenocarcinoma samples and normal tissues, utilizing a diverse array of statistical methodologies, including Student’s *t* test, analysis of variance, and other state-of-the-art approaches, while employing innovative visualization methods such as box plots and scatter plots to vividly showcase its expression pattern and distribution. From TCGA databases, it was found that the levels of *SKA1* in various tumors were significantly higher than those in normal tissues ( Figure 1A,B), with lung cancer emerging as a prime candidate for further exploration due to its uniquely high expression levels of *SKA1* gene. Our analysis revealed that expression of the *SKA1* gene was significantly higher in lung cancer ( *P*<0.05) than that in normal tissues. Moreover, the average expression (median value) of the *SKA1* gene in 539 cases of lung adenocarcinoma was significantly higher than that in 59 normal tissue samples (2.53 vs 0.80; Figure 1C). Similarly, the average expression of *SKA1* in 58 lung adenocarcinoma tissue samples was higher than that in matched paracancerous tissue samples (2.64 vs 0.80; Figure 1D).

The Kaplan-Meier method was used to scrutinize the effect of *SKA1* gene expression on patient survival and progression time, leading to a deeper understanding of the relationship with lung adenocarcinoma. The results of the Kaplan-Meier survival analysis indicated a robust relationship between high levels of SKA1 protein and poor short-term survival outcomes ( Figure 1E–G). Furthermore, we conducted a comprehensive and meticulous evaluation of the correlation between *SKA1* gene expression and various clinical features, ranging from age and sex to TNM stage and tumor location. The results are shown in Table 1. Subsequently, Cox proportional risk model analysis was employed to determine the prognostic value of SKA1 expression levels, revealing it as an independent and detrimental prognostic factor with a statistically significant *P* value of 0.013 and a corresponding 95% confidence interval of 1.549 (1.431‒1.676; Table 2). ROC curve analysis effectively determined its predictive ability as a diagnostic marker of lung adenocarcinoma. After careful analysis of the ROC curve, the *SKA1* gene was determined to be a highly sensitive and specific diagnostic marker for nodular lung adenocarcinoma. The 95% confidence interval for the area under the ROC curve (AUC) was calculated to be 0.928 to 0.969, demonstrating its high diagnostic accuracy. Cut-off values of 1.25, 0.88, 0.93, and 0.82 were determined ( Figure 1H).

**Table:** **Table 1** Clinicopathological characteristics of high and low SKA1 expression groups

Characteristics	Low expression of SKA1	High expression of SKA1	*P* value
Number ( *n*)	322	322	
Thologic T stage, n (%)			0.296
T1	6 (0.9%)	14 (2.2%)	
T2	56 (8.7%)	56 (8.7%)	
T3	220 (34.2%)	218 (33.9%)	
T4	40 (6.2%)	34 (5.3%)	
Pathologic N stage, n (%)			**0.003**
N0	164 (25.5%)	206 (32.0%)	
N1	86 (13.4%)	68 (10.6%)	
N2	72 (11.2%)	48 (7.5%)	
Pathologic M stage, n (%)			**0.036**
M0	249 (38.7%)	270 (41.9%)	
M1	73 (11.3%)	52 (8.1%)	
Pathologic stage, n (%)			**0.003**
Stage I	52 (8.1%)	63 (9.8%)	
Stage II	105 (16.3%)	138 (21.4%)	
Stage III	107 (16.6%)	87 (13.5%)	
Stage IV	58 (9.0%)	34 (5.3%)	
Gender, n (%)			0.937
Female	151 (23.4%)	150 (23.3%)	
Male	171 (26.6%)	172 (26.7%)	
Race, n (%)			0.977
Asian	26 (4.0%)	27 (4.2%)	
Black or African American	83 (12.9%)	81 (12.6%)	
White	213 (33.1%)	214 (33.2%)	
Age, n (%)			0.873
≤65	137 (21.3%)	139 (21.6%)	
>65	185 (28.7%)	183 (28.4%)	
CEA level, n (%)			0.430
≤5	236 (36.6%)	227 (35.2%)	
>5	86 (13.4%)	95 (14.8%)	
Lymphatic invasion, n (%)			**0.033**
No	163 (25.3%)	190 (29.5%)	
Yes	159 (24.7%)	132 (20.5%)

**Table:** **Table 2** Cox analysis of the prognosis of patients with LUAD

Characteristics	Number ( *n*)	Univariate analysis		Multivariate analysis
Hazard ratio (95% CI)	*P* value	Hazard ratio (95% CI)	*P* value
Pathologic T stage	644					
T1	20	Reference				
T2	112	0.429 (0.154‒1.159)	0.105			
T3	438	0.425 (0.160‒1.125)	0.085			
T4	74	0.364 (0.126‒1.051)	0.062			
Pathologic N stage	644					
N0	370	Reference			Reference	
N1	154	0.629 (0.431‒ 0.919)	**0.017**		0.545 (0.341‒0.870)	**0.011**
N2	120	0.531 (0.349‒0.807)	**0.003**		0.579 (0.096‒3.488)	**0.551**
Pathologic M stage	644					
M0	519	Reference			Reference	
M1	125	0.657 (0.442‒0.975)	**0.037**		0.779 (0.137‒4.425)	0.778
Gender	644					
Female	301	Reference				
Male	343	1.013 (0.743‒1.380)	0.937			
Age	644					
≤65	276	Reference				
>65	368	0.975 (0.714‒1.332)	0.873			
Smoker	644					
Yes	475	Reference				
No	169	1.049 (0.739‒1.491)	0.788			
SKA1	644	1.518 (1.408‒1.637)	**<0.001**		1.549 (1.431‒1.676)	**0.013**

Then, we delved deeply into the intricate signalling pathways and molecular mechanisms that underlie the operation of the *SKA1* gene using GO/KEGG and other functional enrichment analyses. The results of GO/KEGG analysis indicated significant upregulation of a grand total of 458 genes and downregulation of 596 genes in lung adenocarcinoma compared with normal tissues according to the standard of a *P* value less than 0.05 and a fold change greater than 2 ( Figure 1I,J). Remarkably, the high expression of the *SKA1* gene in lung adenocarcinoma was intricately correlated with several other pivotal genes and pathways, such as CPTX2, MAGEA4, NTS, HOXD13, and MAGEA9B ( Figure 1K). Among them, it should be noted that the neuroactive ligand-receptor interaction pathway emerged as the most conspicuous signalling pathway for differential gene expression ( Figure 1L).

We utilized the single-sample gene set enrichment analysis (ssGSEA) algorithm to accurately evaluate the degree of immune cell infiltration in TCGA-derived lung adenocarcinoma samples, analyzing the relative abundance of 24 distinct immune cell types, including CD8+ T cells, B cells, and macrophages, to establish correlations between *SKA1* gene mutation and immune cell infiltration levels ( Figure 1M). Figure 1N–U indicates positive correlations between *SKA1* expression and Th2 cells and Tgd cells in lung adenocarcinoma, while negative correlations were observed between SKA1 expression and mast cells and iDCs.

**Figure 2:**
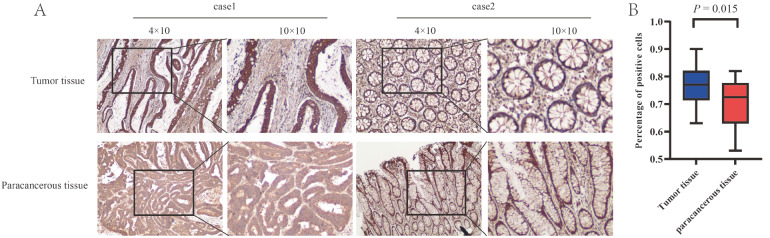
Figure 2 SKA1 expression in lung adenocarcinoma tissues and their matched paracancerous tissues (A) IHC staining of lung adenocarcinoma and paracancerous tissues. (B) Differences in positive cell counts in lung adenocarcinoma and paracancerous tissue.

In this study, the protocol was approved by the Central Hospital of Shaoyang City Ethics Committee (KY 2023-002-21), and written informed consent was obtained from all participants. Tissue samples from patients with lung adenocarcinoma were collected by the Pathology Department of Shaoyang Central Hospital and used as research materials. All samples underwent histological diagnosis and pathological staging, and complete records of clinical features were obtained. Meanwhile, paracancerous tissues were collected as the control group. Twenty lung adenocarcinoma tissues with paraneoplastic tissues, including 11 males and 9 females with a mean age of 62 years, were adopted to perform IHC staining of SKA1. As we expected, upregulation of SKA1 expression was found in 85% (17/20) of the lung adenocarcinomas. Representative images are shown in Figure 2A. We counted the proportion of positively colored cells using the positively colored cell counting method, in which 10 fields of view were randomly observed under a 40× light microscope. The results, as shown in Figure 2B, showed that there was a significant difference in the expression of SKA1 between cancerous and paracancerous tissues ( *P* =0.015).

Many previous studies have demonstrated the important role of SKA1 in the malignant transformation and progression of multiple cancer types, because this gene can regulate various signaling pathways to affect tumor cell growth and metastasis [6]. Increasing clinical evidence has indicated that poor clinical prognosis, tumor invasion, and metastasis are all connected to increased SKA1 expression in a wide variety of cancers, including non-small cell lung cancer, bladder cancer, and hepatocellular carcinoma [ 7– 9]. A previous study revealed that SKA1 confers protection to NSCLC cells against apoptosis induced by cisplatin, thereby resulting in the development of cisplatin resistance in NSCLC cells [5]. Furthermore, SKA1 appears to modulate the ERK1/2 and Akt signaling pathways in NSCLC cells, which play a crucial role in cancer progression [5]. Zhao *et al*. [3] conducted an investigation into the involvement of the *SKA1* gene in adenoid cystic carcinoma, specifically examining its impact on cellular proliferation, invasion, and metastasis. The outcomes of their research demonstrated that the suppression of the *SKA1* gene effectively impeded the growth, migration, invasion, and metastasis of SACC cells. This effect was achieved through the modulation of cell cycle-related protein expression [3]. Consequently, these findings offer novel insights and potential therapeutic targets for the treatment of adenoid cystic carcinoma. Furthermore, these findings align closely with our own results, thereby suggesting that SKA1 represents a crucial candidate for personalized cancer therapy and a unique predictive marker for diagnosis and prognosis. A variety of bioinformatic approaches and experimental methods were adopted to examine the expression pattern and distribution of the *SKA1* gene in lung adenocarcinoma in this study. Meanwhile, the influence on the biological activity of lung adenocarcinoma, as well as its potential application value as a marker for diagnosis and treatment, were also assessed by investigating the relationships between expression and various clinical characteristics and prognosis. Its significance as a pathogenic component and possible marker for lung adenocarcinoma was supported by the dramatic upregulation of the SKA1 protein within clinical cancer tissue samples and the complicated linkage between several signaling pathways and the SKA1 protein. Significantly, abnormal SKA1 protein levels were linked to poorer patient prognosis; thus, this biomarker has great promise for assessing the prognosis of people with lung adenocarcinoma. In addition, the SKA1 gene showed high sensitivity and specificity for the diagnosis of lung adenocarcinoma, suggesting the potential to offer novel insights and approaches for the individualization of lung cancer diagnosis.

Additionally, our study indicates the potential involvement of SKA1 in the neuroactive ligand-receptor interaction pathway, a novel finding in the literature. Nevertheless, prior research has reported the plausible participation of SKA1 in various crucial mechanisms. For instance, it has been demonstrated that SKA1 modulates the ERK1/2 and Akt signaling pathways, thereby regulating the functionality of NSCLC cells [5]. Moreover, in human adenoid cystic carcinoma, SKA1 is implicated in the regulation of the cell cycle and the pathways associated with cell invasion and metastasis [3]. In gliomas, SKA1 is implicated in the modulation of the Wnt/β-catenin signaling pathway, cell cycle regulation, and epithelial-mesenchymal transition (EMT) [10]. In human hepatocellular carcinoma, SKA1 is involved in various pathways, including the Fanconi anemia pathway, homologous recombination pathway, spliceosome pathway, DNA replication, and cell cycle signaling pathway [11]. It is evident that the signaling pathways mediated by SKA1 exhibit variations across different types of cancers. Consequently, our forthcoming research will focus on elucidating the specific mechanism of SKA1 in lung adenocarcinoma.

In summary, this study offers a novel theoretical and empirical foundation for examining the diagnosis and prognosis of lung adenocarcinoma, thereby significantly enhancing our comprehension of these subjects. However, despite the groundbreaking findings of this study, several limitations were encountered during its execution, such as a limited sample size, the necessity for further refinement of experimental conditions and methodology, and the imperative for extensive research and validation.
